# Combination of a fusogenic glycoprotein, pro-drug activation and oncolytic HSV as an intravesical therapy for superficial bladder cancer

**DOI:** 10.1038/bjc.2011.577

**Published:** 2012-01-12

**Authors:** G R Simpson, A Horvath, N E Annels, T Pencavel, S Metcalf, R Seth, P Peschard, T Price, R S Coffin, H Mostafid, A A Melcher, K J Harrington, H S Pandha

**Affiliations:** 1 Oncology, Faculty of Health and Medical Sciences, University of Surrey, Guildford, UK; 2 Targeted Therapy Team, Institute of Cancer Research, London, UK; 3 BioVex Inc, 34 Commerce way, Woburn, MA, USA; 4 North Hampshire Hospital, Basingstoke, UK; 5 Institute of Molecular Medicine, St James's University Hospital Leeds, Leeds, UK

**Keywords:** oncolytic HSV, bladder cancer, micro CT, orthotopic model, fusogenic glycoprotein, prodrug therapy

## Abstract

**Background:**

There are still no effective treatments for superficial bladder cancer (SBC)/non-muscle invasive bladder cancer. Following treatment, 20% of patients still develop metastatic disease. Superficial bladder cancer is often multifocal, has high recurrences after surgical resection and recurs after intravesical live Bacillus Calmette–Guérin. Oncovex^GALV/CD^, an oncolytic herpes simplex virus-1, has shown enhanced local tumour control by combining oncolysis with the expression of a highly potent pro-drug activating gene and the fusogenic glycoprotein.

**Methods:**

*In vitro* fusion/prodrug/apoptotic cell-based assays. *In vivo* orthotopic bladder tumour model, visualised by computed microtomography.

**Results:**

Treatment of seven human bladder carcinoma cell lines with the virus resulted in tumour cell killing through oncolysis, pro-drug activation and glycoprotein fusion. Oncovex^GALV/CD^ and mitomycin C showed a synergistic effect, whereas the co-administration with cisplatin or gemcitabine showed an antagonistic effect *in vitro*. Transitional cell cancer (TCC) cells follow an apoptotic cell death pathway after infection with Oncovex^GALV/CD^ with or without 5-FC. *In vivo* results showed that intravesical treatment with Oncovex^GALV/CD^ + prodrug (5-FC) reduced the average tumour volume by over 95% compared with controls.

**Discussion:**

Our *in vitro* and *in vivo* results indicate that Oncovex^GALV/CD^ can improve local tumour control within the bladder, and potentially alter its natural history.

Bladder cancer is the fourth most common malignancy in men, with an estimated 70 530 new cases and 14 680 deaths in the USA in 2010 (http://www.cancer.gov). Approximately 70% of these patients initially present with superficial bladder cancer (SBC) (Kirkali *et al*, 2005). The standard treatment for patients with SBC is transurethral resection of the bladder tumour, followed by adjuvant intravesical instillations with chemotherapy and/or immunotherapy BCG (Bacillus Calmette–Guérin). The probabilities of recurrence and progression in non-muscle invasive bladder cancer at 5 years after standard treatment range from 31 to 78% (Sylvester *et al*, 2006). These rates illustrate the modest success of currently available treatments and underline the need for improved therapies.

Oncolytic herpes simplex virus (HSV) vectors have shown promising efficacy against a wide variety of malignancies, both *in vitro* and *in vivo* and clinical trials for patients with metastatic colorectal, head and neck, breast, and prostate cancer, melanoma, and glioma have been completed (Kasuya *et al*, 2005). Oncovex^GALV/CD^ is a third generation oncolytic HSV-1 that combines oncolysis with the expression of a highly potent pro-drug activating gene (yeast cytosine deaminase/uracil phospho-ribosyltransferase fusion (Fcy::Fur)) and the fusogenic glycoprotein from gibbon ape leukaemia virus (GALV). ICP34.5 deletion within the backbone of Oncovex^GALV/CD^ result in tumour selective viral replication (Liu *et al*, 2003). ICP34.5 mutants in which both copies of the gene are mutated are incapable of replicating in neurons, but can replicate in and destroy glioma cells *in vitro* and *in vivo* (Andreansky *et al*, 1996). The precise mechanism for growth of ICP34.5 mutants in each tumour type is not fully understood, but it is known from knockout mouse studies that deletions and mutations in PKR and the IFN receptors allow ICP34.5 mutant growth (Leib *et al*, 1999, 2000), and that these mutations and deletions have been found in a number of tumour types (Haus, 2000). This suggests that the deregulation of different and/or multiple pathways in different tumour types allow tumour selective growth of ICP34.5 mutants (review in Simpson and Coffin (2008)). In addition, a deletion of ICP47 increases the antitumour immune response in the presence of HSV and places the Us11 gene under immediate-early promoter control, which enhances growth of HSV ICP34.5 mutants in tumour cells (Liu *et al*, 2003). Previous studies with Oncovex^GALV/CD^ have shown enhanced cell killing and tumour shrinkage (*in vitro* and *in vivo)* within tumours derived from head (and neck), colon, pancreas, lung and glioma tissue (Simpson *et al*, 2006; Price *et al*, 2010; Wong *et al*, 2010). A version of this virus expressing GM-CSF has also shown promising results in clinical phase I-II trials (Hu *et al*, 2006; Kaufman and Bines, 2010) and is currently in clinical phase III for the treatment of melanoma and head and neck.

Bladder cancer is potentially an ideal tumour model for novel therapies because intravesical delivery is able to expose the tumour to high concentrations of virus. In addition, the umbrella cell layer (i.e., the luminal surface of the urothelium) of the bladder is not rapidly dividing and should therefore be resistant to infection and lysis by replication-competent oncolytic viruses, which selectively infect and replicate within rapidly dividing cells. Therefore, we studied the effect of Oncovex^GALV/CD^ in combination with 5-FC/chemo agents on bladder malignancies *in vitro* and *in vivo* in an orthotopic rat bladder cancer model.

## Materials and methods

### Viruses and cell lines

The viruses used in the study were previously described by Simpson *et al* (2006) and constructed. Oncovex^GFP^ (backbone virus) and Oncovex^GALV/CD^ stocks were supplied by BioVex Inc. (Woburn, MA, USA). Human bladder carcinoma cells (EJ, T24, RT112) and baby hamster normal kidney cells (BHK-21) were purchased from American Tissue Culture Collection (ATCC, Manassas, VA, USA). Other human bladder carcinoma cells (VMVUB-I, TCCSUP-G, 5637, KU19-19) were kindly given by Professor Margaret Knowles (Cancer Research UK Clinical Centre, Leeds, UK). The rat bladder carcinoma cell line (AY-27) was kindly given by Dr Ronald B Moore (University of Alberta).

### Fusion assay

The transitional cell cancer (TCC) cells were infected with Oncovex^GALV/CD^ or Oncovex^GFP^ at MOI between 10–0.0001 and incubated at 37 °C for 48 h. Cells were then either fixed and stained with Glutaraldehyde, Crystal Violet, (Sigma, St Louis, MO, USA) or treated with MTS reagent (Promega, Madison, WI, USA).

### Prodrug-activating assay

The TCC cells were infected with Oncovex^GALV/CD^ or Oncovex^GFP^ at MOI between 1–0.01. After 30 min at 37 °C/5% CO_2_, the virus was removed, and full growth media containing 5-FC (C_4_H_4_FN_2_O; Sigma) was added and incubated for 48 h at 37 °C/5% CO_2_. The cell supernatant was transferred into a fresh tube, and the cell debris was removed by centrifuging. The supernatants were added to a fresh tube and heat activated at 60 °C for 10 min. The resulting supernatants were allowed to cool to room temperature and added to test cells. Cells were then either fixed and stained using Glutaraldehyde, Crystal Violet, (Sigma) or treated with MTS reagent (Promega).

### *In vitro* synergy assay

The effect of combination of agents on cell proliferation was assessed by calculating combination index (CI) values using CalcuSyn software (Biosoft, Cambridge, UK). Derived from the median-effect principal of Chou and Talalay, the CI provides a quantitative measure of the degree of interaction between two agents. A CI of 1 denotes an additive interaction, >1 antagonism, and <1 synergy. Experiments were done as described for the *in vitro* survival assay using 4, 2, 1, 0.5 and 0.25 times the calculated ED_50_ of each agent in a constant ratio checkerboard design.

### Determination of cell death

Caspase 3 and 7 activity was detected on EJ cells which were infected with either Oncovex^GALV/CD^ or Oncovex^GFP^ (with or without 5-FC/5-FC metabolites) by Caspase Glo 3/7 reagent (Promega). Apoptotic Z-VAD fmk inhibiter (50 uM) and Necrosis inhibiter (20 mM) Fructose was obtained from Sigma.

### Orthotopic rat bladder tumour model

All procedures were approved by United Kingdom Home Office. Fischer F344 female rats were purchased from B&K Universal or Harlan Ltd. The animals were placed in a supine position and were anesthetised with Isoflurane. The catheter (18-gauge BD Venflon) was inserted into the bladder via the urethra. To facilitate the tumour seeding, the bladder mucosa was damaged by instillation with 0.1N hydrochloric acid followed by a rinse with 0.1N sodium hydroxide for neutralisation. The bladder was washed five times with PBS. A suspension of freshly harvested AY-27 HVEM cells (1.5–2.5 × 10^6^ cells) was then instilled and maintained in the bladder for 1 h. After 1 h, the catheters were removed, and the rats were allowed to void spontaneously.

### Immunohistochemistry for Ki67

Paraffin sections were cut at 4 *μ*m, dewaxed, and rehydrated before being subjected to heat-mediated antigen retrieval in a microwave using citrate buffer (10 mM, pH 6.0). Ki67 (ab16667, Abcam, Cambridge, UK) was diluted 1 : 100 in 1% BSA in PBS and incubated on the sections overnight at room temperature in a humidity chamber. The bound primary antibody was detected using the Vectastain Elite ABC peroxidase system kit (VECTOR Laboratories, Burlingame, CA, USA) followed by DAB detection (DAKO, Glostrup, Denmark). To test the specificity of immunostaining, the primary antibody was omitted. Under this condition, no staining was identified.

All animal studies were approved by the University of Surrey ethical review board.

## Results

### Human bladder TCC cell lines are sensitive to viral HSV oncolysis, which is enhanced by the expression of GALV glycoprotein

**Figure 1:**
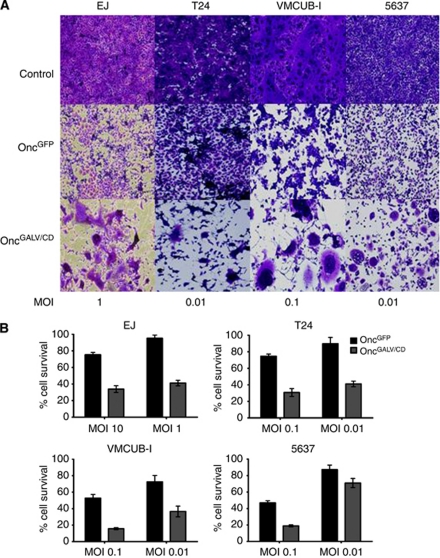
Fusogenic glycoprotein cytotoxic killing of bladder TCC cells. EJ, T24 VMCUB-I and 5637 cells were infected with Oncovex^GFP^ or OncoVex^GALV/CD^ at various MOIs and incubated at 37 °C/5% CO_2_ for 48 h, then assayed by crystal violet staining (**A**) or MTS assay (**B**) (Promega). Average cell survival was calculated as a percentage compared with untreated cells (Unpaired student *t*-test comparing GFP Vs GALV/CD *P*-values: EJ (MOI 10 *P*<0.000, MOI 1 *P*<0.000), T24 (MOI 0.1 *P*<0.000, 0.01 *P*<0.000), VMCUMB-I (MOI 0.1 *P*<0.000, 0.01 *P*<0.000), 5637 (MOI 0.1 *P*<0.000, 0.01 *P*<0.000).

A panel of seven TCC cell lines were tested for viral HSV oncolysis. High viral replication of the oncolytic HSV (Oncovex^GALV/CD^) was observed in all seven TCC cell lines with viral plaque detected as low as 0.1–0.001 MOI. This HSV viral replication led to a strong tumour cytotoxcity effect, which was detected by MTS assay at an MOI as low as 0.001 (data not shown). The expression of GALV glycoproteins enhanced this viral tumour selective killing in four out of the seven TCC cell lines infected with Oncovex^GALV/CD^. Gibbon ape leukaemia virus expression led to the formation of multinucleated syncytia, which were then surrounded with cells showing the more classic HSV-1-mediated effect, (EJ cells, T24 cells, VMCUB-I cells, and 5637 cells) (Figure 1A). To study whether the formation of multinucleated syncytia increased the cytopathic effect of this virus (Oncovex^GALV/CD^) when compared with the backbone virus (Oncovex^GFP^), *in vitro* MTS assays were carried out. Lower levels of MTS activity were seen with OncoVEX^GALV/CD^ than Oncovex^GFP^ on infected EJ (42–54% decrease in cell survival, *P*<0.000), T24 (35–45%, *P*<0.000), VMCUB-I (36–37%, *P*<0.000), and 5637 (35%, *P*<0.000) cells. This suggests that the presence of GALV *env* R-increased tumour cell killing (Figure 1B).

### Fcy::Fur expression converts 5-FC to 5-FU metabolites within human bladder TCC cell lines *in vitro*

**Figure 2:**
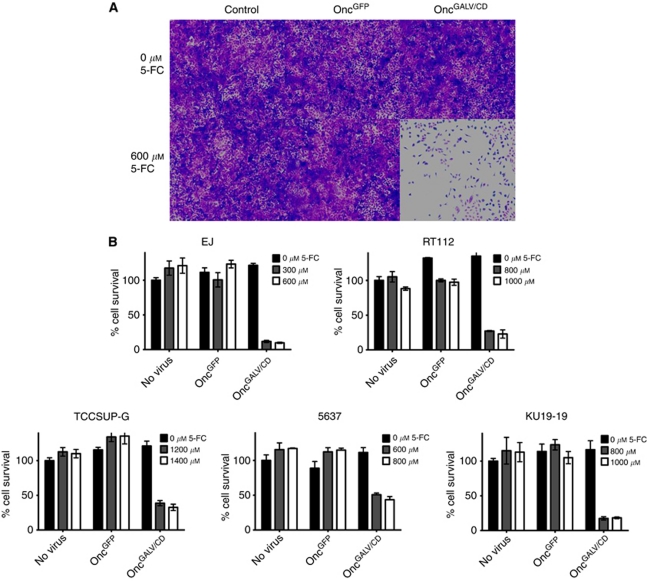
Prodrug activation in TCC cells infected Oncovex^GALV/CD^. EJ, RT112, TCCSUP-G, 5637, and KU19-19 cells were infected with Oncovex^GFP^ and Oncovex^GALV/CD^ at MOI of 0.1 and no virus control. After 30 min at 37 °C/5% CO_2_, the virus was removed, and 1 ml of FGM containing 5-FC (C_4_H_4_FN_2_O; Sigma) at different concentrations (0-1400  *μ*mol l^–1^) was added and incubated for 48 h at 37 °C/5% CO_2_. The cell supernatants were then heat inactivated and added to 1 x 10^4^ fresh target cells and incubated at 37 °C/5% CO_2_ for 72 h and assayed by fixing and staining with glutaraldehyde/crystal violet (**A**) or MTS assay (Promega) (**B**). Average cell survival was calculated as a percentage compared with untreated cells (unpaired student *t*-test comparing GALV/CD+or - 5-FC *P*-values: EJ (300 *μ*mol *P*<0.000, 600 *μ*mol *P*<0.000), RT112 (800 *μ*mol *P*<0.000, 1000 *μ*mol *P*<0.000), TCCSUP-G 1200 *μ*mol *P*<0.000, 1400 *μ*mol *P*<0.000), 5637 800 *μ*mol *P*<0.000, 1000 *μ*mol *P*<0.000), KU19-19 (800 *μ*mol *P*<0.000, 1000 *μ*mol *P*<0.000).

Fcy::Fur is a fusion of two yeast genes *CD* and *UPRT*, which metabolises 5-FC more efficiently than either gene alone. To study the cell killing effects of 5-FC metabolites on TCC cells, we infected human EJ cells with Oncovex^GALV/CD^ or Oncovex^GFP^ in the presence or absence of 5-FC for 48 h at 37 °C. The cell supernatants were then heat inactivated to neutralise the virus and added to fresh EJ cells for a further 72 h at 37 °C. In the presence of supernatants from EJ cells infected with Oncovex^GFP^ no cell death was seen with or without 5-FC (Figure 2A). However, in the presence of supernatant cells exposed to both Oncovex^GALV/CD^ +5-FC, effective cell killing was seen (Figure 2A). Results were similar in a range of human bladder tumour cell lines, including RT112 cells, TCCSUP-G cells, 5637 cells, KU19-19 cells (data not shown). MTS assays were used to quantitate the effects of prodrug activation therapy on bladder tumour cells *in vitro*. EJ cells were the most susceptible to prodrug activation therapy (Figure 2B). A 55% decrease in tumour cell survival was detected on EJ cells at the concentration of 100 *μ*mol 5-FC (Figure 2B), which decreased further to 78% at 600 *μ*mol 5-FC (*P*<0.000) (Figure 2B). RT112 and KU19-19 cells showed moderate sensitivity for Oncovex^GALV/CD^ prodrug activation therapy. In between 600–1000 *μ*mol 5-FC, these cell lines showed around 70% decrease in tumour cell survival (Figure 2B). Finally, TCCSUP-G and 5637 cells showed the lowest sensitivity for this prodrug activation therapy, around 53% decrease in tumour cell survival at 1000 *μ*mol 5-FC (1000 *μ*mol, *P*<0.000) (Figure 2B). From these results, we concluded that five out of seven TCC cells were sensitive to metabolites of 5-FC after infection with Oncovex^GALV/CD^ (in the presence of 5-FC) (Figure 2B).

### Oncovex^GALV/CD^ and chemotherapeutic agent mitomycin C (MMC), show synergistic interaction on bladder TCC tumour cell lines, but not with cisplatin or gemcitabine

Currently, the three mostly clinically used intravesical chemotherapy agents for bladder cancer are MMC, cisplatin, and gemcitabine. The effect of a combination of Oncovex^GALV/CD^ and chemotherapy on cytotoxicity was therefore assessed for significance by isobologram analysis by calculating CI values (see Materials and Methods). Chemotherapy and Oncovex^GALV/CD^ were administered to cells concomitantly. We tested TCC cells including EJ, T24, TCCSUP-G, and KU19-19. We observed synergistic cell killing with Oncovex^GALV/CD^ and MMC on EJ (ED_50_ 0.77±0.05), T24 (ED_50_ 0.65±0.07) and KU19-19 (ED_50_ 0.78±0.01) TCC cells (Table 1). However, a combination of Oncovex^GALV/CD^ and cisplatin/gemcitabine was antagonistic on EJ, (or T24, or TCCSUP-G) cells at most ED_50−90_. An exception to this was Oncovex^GALV/CD^ and gemcitabine on T24 cells at high dose (Table 1). This high dose synergy with Oncovex^GALV/CD^ and gemcitabine was not seen on two other TCC cell lines (EJ and KU19-19, Table 1). The results indicate that the co-administration of Oncovex^GALV/CD^ and MMC showed a synergistic effect, whereas the co-administration with cisplatin or gemcitabine showed an antagonistic effect *in vitro*.

### HSV replication is enhanced by the expression of GALV glycoprotein on TCC cells

**Figure 3:**
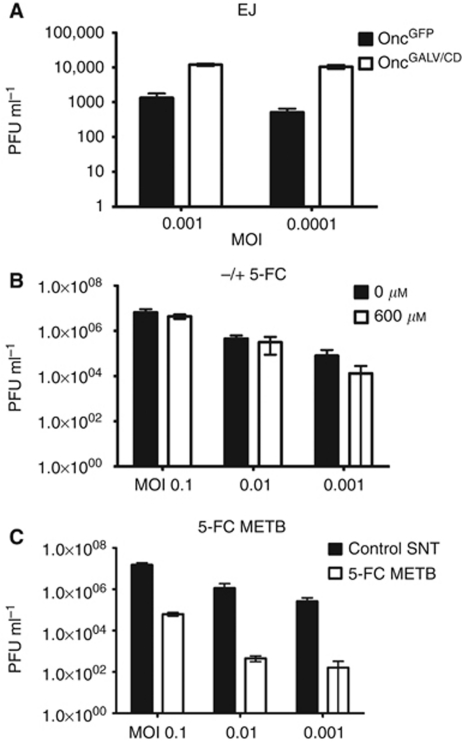
Oncolytic HSV viral replication in the presence of viral glycoprotein, 5-FC and metabolites of 5-FC. (**A**) EJ cells were infected at various MOI (0.001 0.0001) with either Oncovex^GFP^ or Oncovex^GALV/CD^ and incubated at 37 °C for 48 h. Unpaired student *t*-test comparing GFP *vs* GALV/CD *P*-value (MOI 0.001) 0.000 (MOI 0.0001) 0.0002. (**B**) EJ cells were infected at various MOIs (0.1, 0.01, 0.001) with Oncovex^GALV/CD^ in the presence or absence of 5-FC and incubated at 37 °C for 48 h. + or – 5-FC *P*-value (MOI 0.1) 0.03 (MOI 0.01) 0.414 (MOI 0.001) 0.075. (**C**) EJ cells were infected at various MOIs (0.1, 0.01. 0.001) with Oncovex^GALV/CD^ in the presence or absence of metabolites of 5-FC and incubated at 37 °C for 48 h. The resulting virus stocks were titred on BHK cells using a standard plaque assay + or – metabolites of 5-FC *P*-value (MOI 0.1) 0.005 (MOI 0.01) 0.004 (MOI 0.001) 0.0001.

To address whether GALV-related fusion affected HSV replication in TCC cells, Oncovex^GALV/CD^ and control Oncovex^GFP^ virus stocks were prepared on EJ cells. The resulting stocks were replated onto BHK cells, which do express the Pit-1 receptor that allows GALV-related fusion (Sommerfelt and Weiss, 1990). The results show that infection of EJ cells with Oncovex^GALV/CD^ showed a log higher enhanced viral replication compared with control virus (Figure 3A, *P*=0.000). This confirms previous results seen in fibrosarcoma (HT1080) (Simpson *et al*, 2006). We predict that the process of fusion allows more cellular resources to be obtained, which aids viral replication.

### HSV replication on TCC cells is not inhibited by 5-FC but can be inhibited by 5-FC metabolites

The conversion of 5-FC to 5-FU results in an inhibition of host DNA replication that might be expected to also inhibit HSV. This was investigated on EJ cells by infecting them with Oncovex^GALV/CD^ at various MOI with or without 600 mM 5-FC. The resulting infected lysates were then titred on BHK cells and showed no significant difference in titre with or without 5-FC, Figure 3B. In contrast, if EJ cells were exposed to the metabolites of 5-FC at the point of infection with Oncovex^GALV/CD^ a dramatic drop in viral replication was seen over 10-fold (Figure 3C, *P*-value (MOI 0.1) 0.005). Our results suggest that primary oncolytic HSV replication is not inhibited by the conversion of 5-FC to 5-FU by Fcy::Fur in EJ cells, but subsequent rounds of infection may be.

### TCC cells follow an apoptotic cell death pathway after infection with Oncovex^GALV/CD^ with or without 5-FC

**Figure 4:**
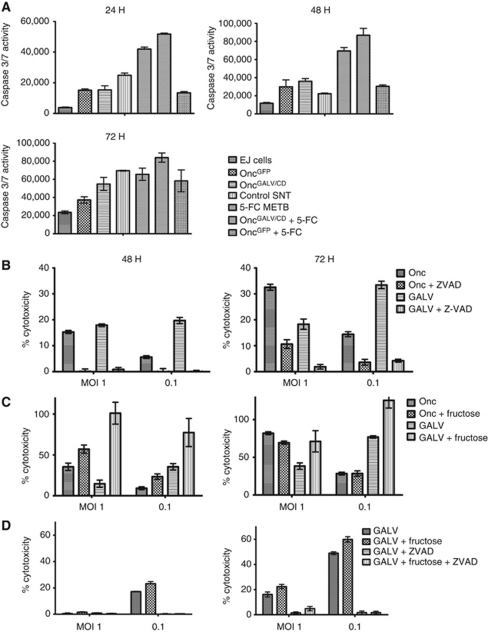
Bladder TCC cells follow apoptotic pathway after infection with Oncovex^GALV/CD^. (**A**) EJ cells were infected with Oncovex^GFP^ or Oncovex^GALV/CD^ (MOI 1) with or without 5-FC (600 *μ*M) or its metabolites for 24, 48, and 72 h. Caspase activity was detected using Caspase Glo 3/7 reagent (Promega). Unpaired student *t*-test comparing GFP *vs* GALV/CD *P*-value 24 h 0.8919 48 h 0.2596 72 h 0.061. (**B**) EJ cells were infected Oncovex^GALV/CD^ or Oncovex^GFP^ at MOIs of 1, 0.1 and then incubated with and without 50 uM Z-VAD-FMK (Sigma) for 48/72 h. After which, the supernatant of the samples were assayed for lactate dehydrogenase activity (LDH) a marker of cytotoxic cell death (Roche). (**C**) EJ cells were infected with Oncovex^GALV/CD^ or Oncovex^GFP^ at MOIs of 1, 0.1 and then incubated with and without 20 mM fructose and incubated for 48/72 h at 37 °C. LDH assays were carried out on sample supernatant. (**D**) EJ cells were infected with Oncovex^GALV/CD^ at MOIs of 1, 0.1 and then incubated with either 50 *μ*M Z-VAD-FMK or 20 mM fructose or both and incubated/assayed as above (unpaired student *t*-test comparing *P*-value Onc *vs* Onc ZVAD, 48 h MOI 1 *P*>0.0000, MOI 0.1 P 0.0127, 72 h MOI 1 *P*>0.0000, MOI 0.1 P 0.000. GALV *vs* GALV ZVAD 48 h MOI 1 *P*>0.0000, MOI 0.1 *P*>0.0000, 72 h MOI 1 *P*>0.0001, MOI 0.1 *P*>0.0000. Onc *vs* Onc Fru, 48 h MOI 1 P0.0078, MOI 0.1 P 0.0032, 72 h MOI 1 0.0051, MOI 0.1 0.3229, GALV *vs* GALV Fru 48 h MOI 1 *P*>0.0015, MOI 0.1 *P*>0.0001, 72 h MOI 1 0.0595, MOI 0.1 0.0148).

Transfection of viral fusogenic membrane GALV in tumour cells results in non apoptotic death (Bateman *et al*, 2000; Higuchi *et al*, 2000; Galanis *et al*, 2001; Linardakis *et al*, 2002; Lin *et al*, 2010). Apoptosis is triggered early in a HSV-1 infection. During the later stages of the virus life cycle, anti-apoptotic proteins such as ICP4, ICP6, US11, gD, and gJ (Nguyen and Blaho, 2007) are produced allowing the cells to assemble viral progeny before apoptotic cell death (Nguyen and Blaho, 2007). The expression of CD-UPRT in the presence of 5-FC also results in apoptotic death within tumour cells (Gopinath and Ghosh, 2008). In our study, apoptotic death was measured in EJ cells by measuring caspase activity. EJ cells were infected with Oncovex^GALV/CD^ or Oncovex^GFP^ with or without 5-FC/5-FC metabolites and caspase 3 and 7 activity were measured using caspase Glo 3/7 reagent (Promega). At the early time point (24 h), both Oncovex^GALV/CD^ and Oncovex^GFP^ infection of EJ cells showed limited caspase 3 and 7 activity compared with cells exposed to 5-FC or 5-FC metabolites (Figure 4A). At later time points, the caspase signal in HSV-infected cells rose in comparison with cells exposed to prodrug therapy (Figure 4A), suggesting that the caspase assay has not picked up viral-driven early apoptosis owing to the fact that the virus has got through more than one round of replication at 24 h and that anti-apoptotic viral proteins inhibited its signal until the later stage of the viral life cycle. It was interesting to see that Oncovex^GALV/CD^ + 5-FC produced a caspase signal similar to the 5-FC metabolites, which suggests that early prodrug therapy induced caspase activity as much as its later metabolites (Figure 4A). In addition, viral anti-apoptotic proteins, failed to inhibit prodrug-induced caspase activity (Figure 4A) and this early prodrug-induced caspase activity did not inhibit viral replication (Figure 3B). When comparing caspase activity between the oncolytic HSV expressing GALV glycoprotein and the backbone virus, no difference was seen at the early time points (24, 48 h, Figure 4A), a slight increase was seen with GALV expression at 72 h, but this was not statistically significant (72 h, Figure 4A, *P*=0.06). It was concluded that GALV glycoprotein expression did not alter caspase activity in infected EJ cells.

### Caspase inhibiter blocks Oncovex^GALV/CD^ cell death pathway in bladder TCC cells *in vitro*. In contrast, a necrosis inhibiter fails to inhibit such cell death

To establish whether Oncovex^GALV/CD^ cell death is a purely apoptotic-driven process, infected EJ cells were exposed to Z-VAD fmk (a pan caspase inhibiter), after which samples were assayed for lactate dehydrogenase activity, a marker for cytotoxic cell death. Results showed that in EJ cells infected either with Oncovex^GALV/CD^ or Oncovex^GFP^, cytotoxic cell death can be blocked almost totally by inhibiting the caspase cascade (Figure 4B), suggesting that both viruses cause cell death by an apoptotic pathway. Apoptosis is mediated by caspase protease activation and requires ATP (Rosser and Gores, 1995; Nicotera *et al*, 1998; Bernardi *et al*, 1999). Necrosis results from ATP depletion (Rosser and Gores, 1995; Green and Reed, 1998). Adding fructose to cells generates ATP through glycolysis, which is sufficient to inhibit necrotic cell death (Anundi and de, 1989). To investigate this necrotic component, EJ cells were infected with Oncovex^GALV/CD^ or Oncovex^GFP^ and incubated for 24/48 h. Our results indicate that the presence of fructose enhanced cell death rather than inhibiting it in EJ cells infected with Oncovex^GALV/CD^ (and to a lesser extent in Oncovex^GFP^) (Figure 4C). The positive control for this experiment was Ionomycin that cause primary necrosis (Gwag *et al*, 1999), which can be rescued by 20 mM fructose (data not shown). This suggests that the cytotoxicity was controlled by a non-necrotic pathway and that the fructose was feeding the apoptotic pathway. This hypothesis was confirmed by the fact that fructose enhances cell death and can be blocked by Z-VAD fmk (Figure 4D). Taken as a whole, this data suggests that Oncovex^GALV/CD^ cell death is a purely apoptotoic-driven pathway rather than through necrosis/autophagy as seen during transfection of viral fusogenic membrane GALV (Lin *et al*, 2010).

### Treatment of a rat orthotopic bladder tumour model with Oncovex^GALV/CD^ with or without 5-FC

**Figure 5:**
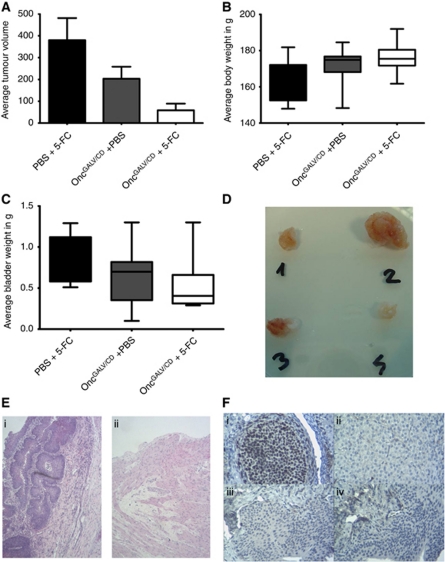
Treatment of a rat orthotopic bladder tumour model with Oncovex^GALV/CD^ +5-FC. AY-27 HVEM cells were implanted into the bladders of female Fischer F344 rats at day 0. The tumour-bearing animals were assigned into three treatment groups either Oncovex^GALV/CD^+5-FC (*n*=10), Oncovex^GALV/CD^ +PBS (*n*=10) or PBS+5-FC (control group) (*n*=8). Intravesical treatment of implanted tumours was carried out with virus (Oncovex^GALV/CD^, 9e7 pfu) or control PBS on days 7, 14, and 21. Prodrug 5-FC (12.5 mg ml^–1^) or PBS was installed in the same manner on days 8, 9, 15, 16, 22, and 24 and the animals were killed on day 28. Their bladders were removed and assessed for tumour abundance. The harvested bladders were weighed, then opened up and the bladder surface, which macroscopically contained tumour was measured with a caliper. Average tumour volume was calculated by measuring length x width/2. (**A**) Average tumour volume (**B**) Average body weight (**C**) Average bladder weight (unpaired student *t*-test comparing tumour volume PBS *vs* GALV/CD +5-FC *P*=0.001, PBS GALV/CD- 5-FC *vs* GALV/CD +5-FC *P*=0.034, PBS *vs* GALV/CD −5-FC *P*=0.13. (**D**) Bladder seeded with tumour cells (no treatment) and removed at necropsy after 18 days (1, 2 and 3). Bladder 4 was a control in which bladder mucosa was conditioned (acid/alkali rinse) but no cells were seeded. (**E**) H&E stain of sections from bladder from animals A (i) and B (ii). (**F**) Ki67 stain (brown) of sections from bladder from animals A (i) and B (iii). No primary antibody control sections from animals A (ii) and B (iv).

To model *in vivo* human SBC, a rat orthotopic bladder tumour model was used that was previously described by Xiao *et al* (1999). AY-27 cells are a rat bladder transitional cell carcinoma cell line, which was established as a primary bladder tumour in Fischer f344 rats, which were feed FANFT (N(4-(5-Nitro-2-Furyl)-2-Thiazolyl Formamide) (Xiao *et al*, 1999). Histological examination of the *in vitro* and *in vivo* tumour specimens confirmed the presence of grade II-III transitional cell carcinoma (Xiao *et al*, 1999). AY-27 cells, which were previously tested for susceptibility for HSV replication were used in this model. AY-27 cells were stably transfected with the herpes virus entry receptor (HVEM) and a clone selected that supported infection with HSV. Fusion assay results showed a reduction in metabolic enzyme activity of up to 30% (MTS assay) in AY-27 HVEM cells infected with Oncovex^GALV/CD^ compared with the Oncovex^GFP^ control (Supplementary Figure S1). This is in addition to the cytotoxcity seen with HSV replication alone. AY-27 HVEM cells were further tested in our prodrug assay, which showed that Oncovex^GALV/CD^ can metabolise 5-FC within these cells, resulting in a decrease in metabolic enzyme activity of up to 81% (MTS assay) when compared with controls (Supplementary Figure S1). To facilitate tumour seeding, the bladder mucosa was conditioned with an acid rinse followed by neutralisation with alkali. After tumour seeding, a high success rate of implantation was seen (>95%). Figure 5D shows an example of such an *in vivo* experiment (without treatment) where tumours were seeded and the bladder removed at necropsy after 18 days. The first three bladders showed a high tumour load (Figures 5D, 1, 2 and 3). The fourth bladder was a control in which bladder mucosa was conditioned (acid/alkali rinse) but no cells were seeded (Figure 5D, 4). The tumour implantation procedure was well tolerated by the animals. To study the effects of Oncovex^GALV/CD^ *in vivo,* freshly harvested AY-27 HVEM cells were installed as described above (day 0). The tumour-bearing animals were assigned into three treated groups either Oncovex^GALV/CD^+5-FC (*n*=10), Oncovex^GALV/CD^ +PBS (*n*=10) or PBS+5-FC (control group) (*n*=8). Intravesical treatment of implanted tumours was carried out with virus (Oncovex^GALV/CD^, 9e7 pfu) or control PBS on days 7, 14, and 21. Prodrug 5-FC (12.5 mg ml^–1^) or PBS was installed in the same manner on days 8, 9, 15, 16, 22, and 24 and the animals were killed on day 28. Bladders were removed and assessed for tumour growth. The results showed an 84.5% reduction in average tumour volume in the presence of both Oncovex^GALV/CD^ and prodrug when compared with control (*P*=0.001) or virus alone *P*=0.034) (Figure 5A). A smaller amount of tumour shrinkage seen with virus alone was not statistically significant when compared with control animals (46.4% tumour reduction), (*P*=0.13) (Figure 5A). In the control groups, no spontaneous tumour regression was seen. The bladders were weighed and showed a trend of lower bladder weight after treatment with both agents (Figure 5C). On average, the animals treated with Oncovex^GALV/CD^ +5-FC were 11.5 g heavier than controls, suggesting that they were in a healthy condition compared with controls (Figure 5B). The results strongly suggest that a combination of oncolysis, prodrug activation and fusogenic glycoprotein therapy offers an opportunity for improved tumour control within the bladder.

### Computed microtomography (microCT) imaging of an orthotopic bladder tumour model, during treatment with Oncovex^GALV/CD^ +5-FC

**Figure 6:**
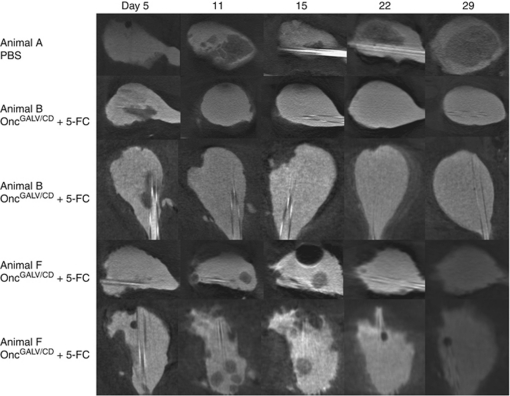
Computed tomography imaging of an orthotopic bladder tumour model during treatment with Oncovex^GALV/CD^ + 5-FC. Intravesical treatment of implanted tumours was carried out with virus (Oncovex^GALV/CD^, 9e7 pfu) (or Control PBS) on days 6, 12, and 18. Prodrug 5-FC (12.5 mg ml^–1^) was installed in the same manner on days 7, 8, 13, 14, 19, and 20 and the animals were killed on day 29. During the experiment, computed tomography imaging was carried out on days 5, 11, 15, 22, and 29.

For the orthotopic *in vivo* experiments, there was no way of establishing how much tumour was present until autopsy. An attempt was made to quantify tumour load by HVEM qPCR in urine of implanted rats. The results showed no reliable signal was detected that correlated to the amount of tumour present at autopsy (data not shown). As a result, tumour growth was evaluated by non invasive imaging using IVIS, by inserting the insect luciferase gene into AY-27 HVEM cells. When these cells were assessed *in vivo* (Fischer F344 flank model), a luciferase signal was detected initially but faded due to regression of the tumour (data not shown). To overcome this problem, microCT was used with the aim of establishing the feasibility of imaging the tumours non-invasively while they were growing and the process of oncolysis/prodrug activation within the bladder of Fischer F344 rats. These rats were implanted with freshly harvested AY-27 HVEM cells. On day 5 (Figure 6) after implantation, a catheter was inserted into the bladder via the urethra with the use of an 18-gauge plastic intravenous cannula (BD Venflon Pro, Oxford, UK), which had been prefilled with iodine contrast (50% iodine contrast in PBS). The bladder was filled with iodine contrast solution and then the animal was scanned on a SkyScan1076 *in vivo* micro CT (Scan parameter: 35 mcm per slice, 68 kV, range of detector motion 192 degrees, 0.5 mm aluminium filter). Further scans were carried out on day 11, 15, 22, and 29 using the same method (Figure 6). Intravesical treatment of implanted tumours was carried out with virus (Oncovex^GALV/CD^, 9e7 pfu) (or control PBS) on days 6, 12, and 18. Prodrug 5-FC (12.5 mg ml^–1^) was installed in the same manner on days 7, 8, 13, 14, 19, and 20 and the animals were killed on day 29. Tumours were observed in animal A, which had received no virus treatment (PBS)+5-FC. This animal can be described as having disease progression, which filled the bladder with tumour over the course of the experiment (Figure 6). The presence of a large amount of tumours was confirmed by histology using H&E stain (Figure 5E i). The tumour was also shown to be proliferating using Ki67 staining (Figure 5F i). Animals B and F received Oncovex^GALV/CD^ +5-FC treatment as described above. Animal B had similar initial tumour load to animal A and showed a complete response of pedunculated intraluminal disease (Figure 6). Histology (H&E stain) on this animal confirmed that almost no tumour was present after treatment (Figure 5E ii). In the only small area of disease identified in animal B, no proliferating tumour cells were detected by Ki67 staining confirming a pathological complete response. Animal F had a much higher initial tumour load than both A or B at the start point of treatment, but still showed a partial response in intraluminal pedunculated disease (Figure 6). The results clearly show that it is possible to visualise the growth of tumours and the process of oncolysis/prodrug activation in an orthotopic bladder tumour model.

## Discussion

Surgery, BCG and chemotherapy dramatically slow the progress of bladder cancer, but do not eradicate the disease totally (Sylvester *et al*, 2006). BCG therapy is the reference standard in non-surgical treatment for high-risk non-muscle invasive bladder cancer but is associated with relatively high morbidity, with up to 25% of patients failing to complete a full maintenance course. Clearly, there is a need to develop new treatment strategies for bladder cancer, particularly for high-risk superficial tumours with BCG treatment failure and muscle invasive cancer.

In our study, we have shown that this viral-based triple therapy offers a novel approach to intravesical treatment of bladder cancer. Our choice of an oncolytic HSV, based on experience with Oncovex^GALV/CD^, has been found to be effective in treating various experimental cancers while maintaining an excellent safety profile (Simpson *et al*, 2006; Price *et al*, 2010; Wong *et al*, 2010).

The panel of cell lines were selected for the study because they represent human bladder cancer with RT112 cells specifically reflecting non-muscle invasive disease. All human bladder tumour cell lines tested (*in vitro*) were susceptible to HSV oncolysis and showed enhanced tumour cell killing with either fusion or prodrug therapy (or both) when infected with Oncovex^GALV/CD^ virus. Fusion therapy could enhance HSV oncolysis by decreasing tumour TCC cell survival by up to 54%. Prodrug therapy from Oncovex^GALV/CD^, also killed (bystander effect) uninfected TCC by up to 78%. A synergistic interaction was detected between Oncovex^GALV/CD^ and the chemotherapy agent MMC but not Cisplatin and Gemcitabine. Why is oncolytic HSV synergistic with MMC but not cisplatin/gemcitabine on bladder TCC? Oncolytic HSV-1 viruses have previously been shown to be synergistic with MMC, (a potent cross-linker of DNA) on one bladder TCC cell line (Mullerad *et al*, 2005) and a number of gastric cancer cell lines (Bennett *et al*, 2004). In contrast, gemcitabine has previously been shown to have limited synergy with HSV in pancreatic cancers cell line, but this interaction does inhibit viral replication (Watanabe *et al*, 2008). MMC is one of the range of DNA-damaging agents, which induces the expression of GADD34 (growth arrest and DNA damage –inducible protein) (review in Kanai *et al*, 2010). There is a great homology between the carboxyl terminus of the mammalian GADD34 gene and the corresponding carboxyl domain of the viral ICP34.5/ virulence factor (Chou and Roizman, 1994), deletion of which allows tumour selective viral replication (Rampling *et al*, 2000). RNAi targeting of GADD34 decreased MMC -associated enhancement of HSV replication on gastric cancer cell lines (Bennett *et al*, 2004). This would suggest that MMC may upregulate GADD34 in HSV-infected TCC cells, resulting in synergy between the two therapies. On this basis, we would expect cisplatin to show synergy with HSV in bladder TCC because cisplatin has been shown to upregulate GADD34 in malignant mesothelioma and head/neck squamous carcinoma cell lines (Adusumilli *et al*, 2006; Fishel *et al*, 2006). Our results do not show synergy between cisplatin and oncolytic HSV on bladder TCC cells. Therefore, we can hypothesise that the cellular components needed for upregulation of GADD34 by cisplatin are present in malignant mesothelioma and head/neck squamous carcinoma cell lines but may not be present in bladder TCC cells. To study the effect of Oncovex^GALV/CD^ *in vivo,* we further developed an orthotopic rat bladder model and confirmed safety, efficacy, and ease of delivery of oncolytic viral therapy for experimental treatment of bladder cancer. Oncovex^GALV/CD^ virus, when administered with prodrug weekly for 3 weeks by intravesical instillation, was clearly more effective than control or virus alone at reducing tumour burden (84.5% reduction, *P*=0.001). To evaluate the *in vivo* orthotopic tumour growth and then oncolysis, we tried non-invasive imaging including IVIS and CT scanning. Using intravesical iodine contrast and CT scanning, we were able to visualise the growth of tumours and oncolysis following Oncovex^GALV/CD^ treatment.

A range of viral non-replicating vectors and oncolytic viruses have been studied in both mouse and rat orthotopic bladder tumour models. Non-replicating vectors such as lentivirus (Kikuchi *et al*, 2004; Adam *et al*, 2007), adenovirus (Werthman *et al*, 1996; Sutton *et al*, 2000) and poxviruses (Fodor *et al*, 2005) have shown strong therapeutic gene expression in bladder cancer including endostatin (Shichinohe *et al*, 2001) and p53 (Ruifa *et al*, 2006). Oncolytic viruses such as retroviral (RCR) vectors (Kikuchi *et al*, 2007), adenovirus (Wang *et al*, 2006), VSV (Hadaschik *et al*, 2008), reovirus (Hanel *et al*, 2004), vaccinia virus (Fodor *et al*, 2005) and HSV-1 (Cozzi *et al*, 2001) offer bladder tumour selective killing due to viral replication and even stronger therapeutic gene expression due to multiple rounds of replication and promoter upregulation (Hadaschik *et al*, 2008). These viral orthotopic bladder studies have shown some efficacy, but only a few of these viral vectors have gone on to clinical trials. Those that have include adenovirus (Kuball *et al*, 2002; Pagliaro *et al*, 2003; Malmstrom *et al*, 2010) and vaccinia virus (Gomella *et al*, 2001). Strong gene transfer (WT p53, CD40L) has been seen using non replicating adenovirus in patients biopsies, but only one clinical response was seen in these early I/IIa trials (Kuball *et al*, 2002; Pagliaro *et al*, 2003; Malmstrom *et al*, 2010). In contrast, an oncolytic adenovirus expressing GM-CSF (in combination with BCG) showed a response rate of 46% as assessed by cytoscopy and urine cytology or biopsy (McKiernan *et al*, 2009). Further studies have shown three out of four patients treated with an oncolytic vaccinia virus have been shown to be disease free after 4 years (McKiernan *et al*, 2009). HSV-1, like vaccinia, is a large DNA virus, which can be easily manipulated to allow tumour selective virus replication (MacLean *et al*, 1991; Martuza *et al*, 1991; Rampling *et al*, 2000) and allows insertion of therapeutic genes of up to 30 kb (Roizman and Jenkins, 1985). Oncolytic HSV can infect a broad range of human tumour cell types. It is a highly lytic virus, resulting in tumour cell death, but does not integrate into the host genome avoiding the possibility of activating proto-oncogenes.

We refined an existing rat orthotopic bladder tumour model to evaluate the efficacy of the OncovexGALV/CD virus with or without 5-FC. Advantages of a rat versus a mouse model include its size and evidence that the rat model may represent the human disease processes better than the mouse equivalent (Oyasu, 1995). In these models, the traditional endpoints of tumour size are not appropriate as the evolving intravesical tumour may block both ureters and cause urinary obstruction and renal failure. We therefore sought to establish a robust non-invasive method of monitoring response to intravesical therapy. Other assessment modalities have been used including urine cytology (Gronlund-Pakkanen *et al*, 2003), ultrasound (Rooks *et al*, 2001), MRI (Mazurchuk *et al*, 1997), qPCR (Wang *et al*, 2006) and bioluminescence imaging (Ray and Gambhir, 2007). We were able to closely monitor evolving tumour and response to treatment by CT in animals with high and low tumour burdens.

We hope in the future to evaluate the *in vivo* orthotopic tumour growth (and oncolysis), by using bioinformatics analysis of the CT scanning data. We hope to obtain from this data, an accurate measurement of the volume and density of the *in vivo* tumours. The measure of tumour density will give an indication of the presence of necrosis, which will help to establish a truer picture of the efficacy of any oncolytic virus tested. We are negotiating with the company Amgen as to its potential clinical application.

In conclusion, we found Oncovex^GALV/CD^ to be effective *in vitro* and in treating an orthotopic rat bladder cancer. A version of this virus expressing GM-CSF has shown promising results in phase I and II (Hu *et al*, 2006; Senzer *et al*, 2009) with limited toxicity to patients and is currently in phase III clinical trials in melanoma and head and neck cancer. Our results suggest that Oncovex^GALV/CD^ (+ 5-FC) intravesical therapy should be considered for human application.

### Statistics

The values of the experiments were presented as the mean±s.d. (or s.e.) and analysis of variance was measured using unpaired Student's *t*-test. Statistical significance was determined at a *P*-value <0.05.

## Figures and Tables

**Table 1:** (a) Summary of *in vitro* therapy data; (b) Interaction of Oncovex^galv/CD^ and mitomycin C on three TCC cell line; (c) Interaction of Oncovex^galv/CD^ and cisplatin on three TCC cell line; (d) Interaction of Oncovex^galv/CD^ and gemcitabine on three TCC cell line

**Cell type**	**Hystological type**	**HSV-1 CPE efficacy**	**Fusion efficacy**	**Prodrug efficacy**
*(a)*				
EJ	TCC	+	+	+
T24	TCC	+	+	−
RT112	TCC	+	−	+
VMCUB-I	TCC	+	+	−
TCCSUP-G	TCC	+	−	+
5637	TCC	+	+	+
KU19-19	TCC	+	−	+

**Cell line**	**ED_50_**	**ED_70_**	**ED_95_**
*(b)*			
EJ	0.77±0.05	0.80±0.04	0.86±0.04
T24	0.65±0.07	0.61±0.04	0.73±0.07
KU19-19	0.78±0.01	0.58±0.06	0.57±0.08
			
*(c)*			
EJ	2.6±0.12	2.29±0.01	2.11±0.01
T24	2.34±0.02	2.03±0.24	1.8±0.4
TCCSUPG	1.73±0.16	1.28±0.13	1.09±0.21
			
*(d)*			
EJ	2.92±0.38	2.08±0.24	1.53±0.24
T24	1.54±0.43	0.89±0.22	0.59±0.12
TCCSUPG	1.42±0.13	1.18±0.47	1.18±0.78

Abbreviations: CPE=cytopathic effect; HSV=herpes simplex virus; TCC=transitional cell cancer.

The effect of the combination of Oncovex^GALV/CD^ and chemotherapy on cell proliferation was assessed by calculating combination index (CI) values using CalcuSyn software (Biosoft). Derived from the median-effect principle of Chou and Talalay, the CI provides a quantitative measure of the degree of interaction between two or more agents. A CI of 1 denotes an additive interaction, >1 antagonism, and <1 synergy. Experiments were done as described for the *in vitro* survival assay using 4, 2, 1, 0.5, and 0.25 times the calculated ED_50_ of each agent in a constant ratio checkerboard design.
