# Supplementary figures and images for: Combination of a fusogenic glycoprotein, pro-drug activation and oncolytic HSV as an intravesical therapy for superficial bladder cancer
Source: Br J Cancer. 2012 Jan 12;106(3):496–507. doi: 10.1038/bjc.2011.577 (PMC3273343; doi:10.1038/bjc.2011.577)

## Supplementary Figure 1

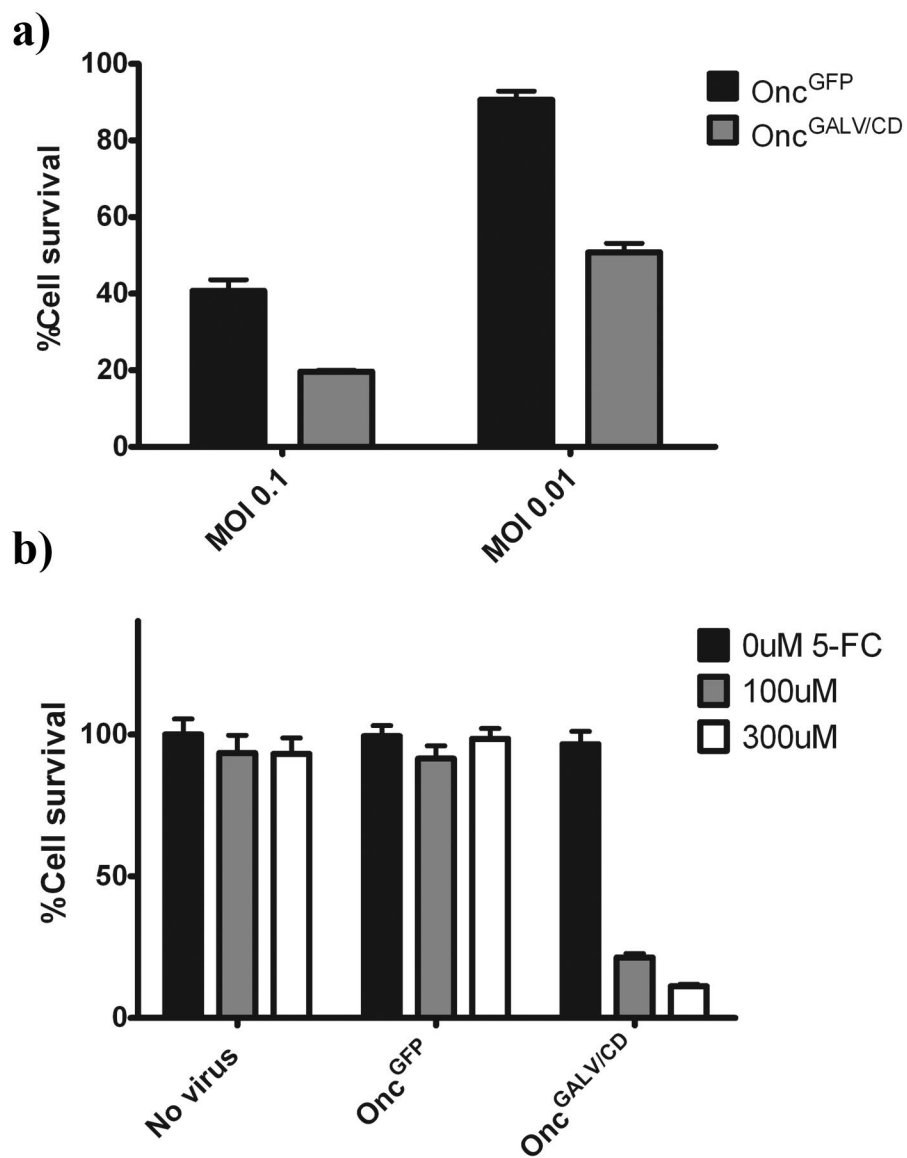

Supplement: Supplementary Figure S1 [file bjc2011577x1.pdf]
