# Supplementary material for: Combination of a fusogenic glycoprotein, pro-drug activation and oncolytic HSV as an intravesical therapy for superficial bladder cancer
Source: Br J Cancer. 2012 Jan 12;106(3):496–507. doi: 10.1038/bjc.2011.577 (PMC3273343; doi:10.1038/bjc.2011.577)
Supplement: Supplementary Figure Legends [file bjc2011577x2.doc]

**Supplementary Figure 1 Fusogenic and Prodrug activation on TCC cells infected Oncovex^GALV/CD^ (+/- 5-FC)**.

**a)** AY-27 HVEM cells were infected with Oncovex^GFP^ or OncoVex^GALV/CD^ at various MOIs and incubated at 37°C/5% CO_2_ for 48 hours, then assayed by MTS assay (Promega). **b)** AY-27 HVEM cells were infected with Oncovex^GFP^ and Oncovex^GALV/CD^ at MOI of 0.1 and no virus control. After 30 minutes at 37°C/5% CO_2_, the virus was removed, and 1ml of FGM containing 5-FC (C_4_H_4_FN_2_O; Sigma) at different concentrations (0-300 µmol/L) was added and incubated for 48 hours at 37°C/5% CO_2_. The cell supernatants were then heat inactivated and added to 1 x 10^4^ fresh target cells and incubated at 37°C/5% CO_2_ for 72 hours and assayed by MTS assay (Promega). Average cell survival was calculated as a percentage compared to untreated cells.
